# Insights from the 2018 Biology of Genomes meeting

**DOI:** 10.1186/s13059-018-1542-x

**Published:** 2018-09-28

**Authors:** Ninad Oak, Sharon E. Plon

**Affiliations:** 1 Departments of Pediatrics and Molecular and Human Genetics, Baylor College of Medicine, Houston, TX USA; 2 Houston, USA

## Abstract

A report on the Cold Spring Harbor Laboratory 31^st^ annual meeting on the Biology of Genomes, held at Cold Spring Harbor, New York, USA, 8–12 May, 2018.

The 2018 Biology of Genomes meeting at Cold Spring Harbor Laboratories covered a wide range of topics with research relevant to many aspects of genomics. Here we highlight two areas of research where multiple talks provided new insights.

## Focusing research away from nucleotide changes in the protein coding genome

In the Cancer and Medical Genomics session, Marcin Imielinski of the New York Genome Center highlighted the enormous complexity of structural rearrangements that can be found in individual cancer genomes. This complexity has challenged current genome presentations and Imielinksi described new tools for the generation of cancer genome graphs (gGraphs) and analysis of complex structural variation (JaBbA -Junction Balance Analysis). Somatic mutation signatures based on single nucleotide variants (SNVs) have been previously described. Specific SNV signature patterns has provided insight to underlying genetic variation and, in some cases, these signatures identify tumors that may be responsive to specific cancer treatments, e.g. *POLE/POLD1* mutations resulting in hypermutation and response to immune therapy. Imielinski’s group presented 10 signatures that were based on the pattern of structural variation within tumors. The method utilized to generate SV signatures combined features derived from classic as well as motif-based patterns of structural variation. The expectation is that these patterns may also subsequently allow recognition of patterns that will implicate specific cancer treatments e.g. homology-directed repair deficiency and response to polyADP ribose polymerase (PARP) inhibition.

Similar to the focus on structural variation as opposed to SNV’s in protein coding genes, there were a number of excellent talks defining variation in non-coding regions from genome sequencing datasets of different patient populations. Patrick Short from Matthew Hurles’ group at the Wellcome Sanger Institute investigated the de novo mutation rate in regulatory elements using over 10,000 whole genome sequencing samples from the Deciphering Developmental Disorders (DDD) study and found that de novo mutations (DNMs) in these individuals are enriched within the ultra-conserved elements and these variants may contribute to 1–3% of subjects without a diagnostic finding. There was substantial overlap between DNMs identified in cohorts diagnosed with developmental delay and autism-spectrum disorders. To facilitate analysis of this type of non-coding variation dataset, the group is now developing a non-coding constraint metric (parallel to the constraint metrics for protein coding genes).

Taking a different approach based on gene expression, Pejman Mohammadi (of the Scripps Research Institute, formerly at the New York Genome Center) presented work utilizing allele-specific gene expression data to identify genetic regulatory outliers in a cohort of patients with muscular dystrophy using Analysis of Expression Variance- Dosage Outlier Test (ANEVA-DOT). These talks and a number of posters at the meeting illustrate our need to develop consistent ways to describe clinically relevant regulatory “grammar”.

The two keynote addresses provided new insights into chromosomal and chromatin regulation in normal and disease states. Wendy Bickmore (University of Edinburgh) described the role of regulatory variation in developmental genes in Mendelian diseases. Bickmore highlighted the central role of chromosomal decompaction in transcriptional regulation as mediated by PARP through phase separation or by Lmbr1 through chromosomal looping.

**Fig. 1:**
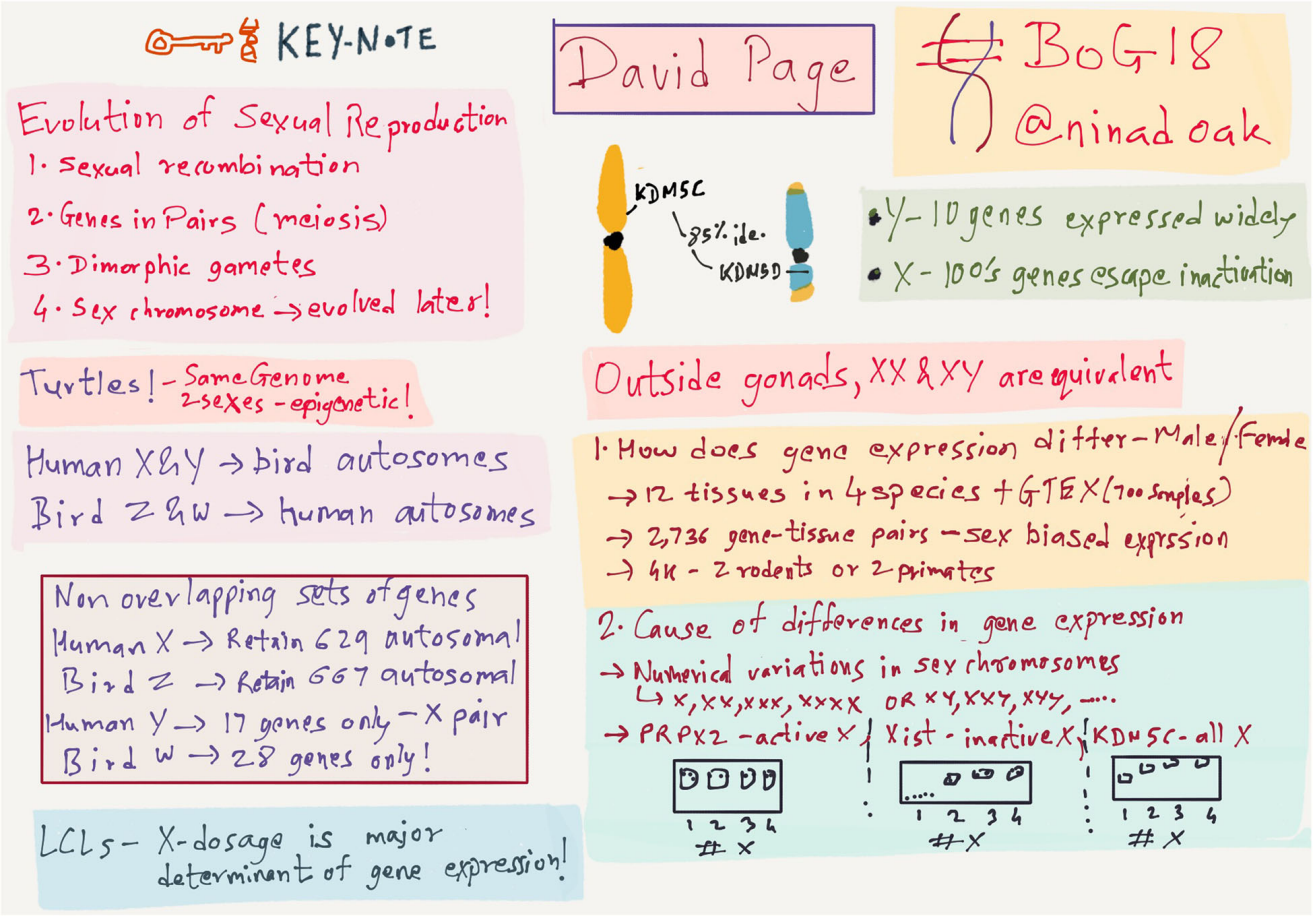
A summary of the presentation by David Page.

In the second keynote address, David Page (Whitehead Institute) investigated overlapping sets of genes retained on sex chromosomes over the course of evolution from autosomes (see Fig. 1). Interestingly, human X chromosome and its bird counterpart Z chromosome retain a much higher number of autosomal genes than the Y and W chromosomes in humans and birds, respectively. Cross-species analysis of autosomal gene expression across 12 tissues identified over 2,700 gene-tissue pairs with conserved sex-biased gene expression. Furthermore, X chromosome dosage is a major determinant of sex-biased autosomal gene expression, potentially explaining the many disorders where penetrance or expressivity may vary between individuals of different sexes.

## New insights from large-scale screens and protein interaction maps

With regard to the “Biology” of genomes, several talks provided early results of elegant, yet massive, screens to better understand the biological and functional consequence of variation seen in either genomic disorders or cancer. Parisa Razaz from Michael Talkowski’s lab at Massachusetts General Hospital modeled isogenic 16p11.2 reciprocal gene disorder using CRISPR mediated deletions and duplications in induced pluripotent stem cells (iPSCs) and mouse models. These models were then used to investigate gene expression differences and the genes which altered expression due to copy number change were enriched in genes expressed at early and late developmental stages and for genes within autism spectrum disorder risk pathways.

Trey Ideker of University of California San Diego described findings from the Cancer Cell Map Initiative (CCMI) designed to generate large protein interaction maps of tumor cells. By integration of somatic RNA expression, whole genome sequencing, and enhancer-gene networks, his group identified 193 somatic eQTLs. Additionally, using hierarchical structured data from yeast, the team developed a deep neural network model (DCell) to predict mechanistic genotype-phenotype relationships giving useful insights into cell structure and function.

Sidi Chen of Yale University presented work from functional cancer genome atlas project that used in vivo AAV-CRISPR screen to reveal a functional map of frequently mutated driver genes in hepatocellular carcinoma and glioblastomas. Gregory Findlay from Jay Shendure’s laboratory at the University of Washington described functional classification of nearly all possible SNVs (~ 4,000 SNVs) in functional domains of *BRCA1* based on CRISPR mediated saturation genome editing. The functional screen identified pathogenic missense variants in *BRCA1* with high accuracy. The increasing use of CRISPR methodologies to facilitate large-scale genetic screens or assist functional assessment of thousands of possible variants within clinically relevant genes is expected to have a high impact on clinical interpretation of variants identified through genetic testing, particularly for rare variants where there is not sufficient patient and/or population related data to determine the pathogenicity of the variant.

## Abbreviations

DNM: de novo mutation
iPSC: Induced pluripotent stem cell
PARP: polyADP ribose polymerase
SNV: Single nucleotide variant

